# Impact of Remote Appointments on the Outcomes of Community Mental Health Nurses in Primary Care Since the Covid Pandemic: A Retrospective Observational Cohort Study

**DOI:** 10.1111/inm.70051

**Published:** 2025-04-24

**Authors:** Mark Kenwright, Paula Fairclough, Charlotte Graham

**Affiliations:** ^1^ University of Staffordshire Centre for Health Innovation Stafford England; ^2^ Keele University Keele England

**Keywords:** Community Mental Health Nurses, post‐pandemic, primary care, remote delivery

## Abstract

The move to remote working during the COVID‐19 pandemic has remained an integral method of mental health service delivery. Yet there is a lack of evidence on the longer‐term impact of this change, or on the comparative effectiveness of different remote formats. This retrospective observational cohort study examined the effect of the move to remote delivery on the effectiveness and practice of Community Mental Health Nurses in primary care. Data from 1634 referrals was examined across three cohorts: Those treated face‐to‐face pre‐pandemic; those treated remotely during the pandemic restrictions; and those treated in a blended approach (remote and face‐to‐face) up to 16 months post‐pandemic. Means, standard deviations and effect sizes for pre–post treatment change are reported for all clinical measures. Logistic regression examined predictors of reliable change. Despite increased severity in the mental health problems treated, effect sizes for remote treatment post‐pandemic (0.5–0.8) were comparable to those for pre‐pandemic face‐to‐face treatment (0.5–0.9). The blended use of online video appointments predicted better engagement and reliable improvement. The sole use of telephone appointments for complex problems predicted lower rates of engagement and improvement.

## 1 Introduction

In response to a worldwide pandemic, most countries implemented strict lockdown measures in 2020 to reduce transmission of the COVID‐19 virus. As in many countries, Community Mental Health Nurses (CMHNs) in the UK implemented rapid and innovative changes to treatment delivery in response to restrictions applied by the UK government (UK Cabinet Office 2020). This necessitated an urgent move to remote treatment delivery, utilising telephone and online video consultations. Although such changes to delivery occurred internationally across most types of healthcare, appointments with Mental Health Nurses (where communication and the therapeutic relationship are central) can seem particularly sensitive to such a radical change in format and delivery method.

Early studies indicated a negative impact of the pandemic on Mental Health Nurses, with many reporting that changes to remote working were implemented too quickly and produced several challenges, such as difficulty engaging service users with remote appointments; increased demand; longer hours and back‐to‐back appointments (Foye et al. 2021). Evidence of initial service user resilience, with a temporary reduction in demand during the early weeks of the pandemic (Rains et al. 2021) was followed by an increase in referrals to mental health services after the first lockdown, which remained at higher levels than before the pandemic after restrictions lifted (Kelleher et al. 2022; Sampson et al. 2022).

In 2019, approximately 970 million people, or 1 in 8 people globally, were living with a mental illness (Institute of Health Metrics and Evaluation 2024). Data indicates a deterioration in mental health across populations during the pandemic, particularly for women, those in lower socio‐economic groups (Patel et al. 2022), adolescents (Marin et al. 2023) and those with a prior history of mental health problems (Fineberg et al. 2021). Although levels of distress lessened, they remained at levels above those prior to the pandemic (World Health Organisation 2022). Internationally, many studies report long‐term detrimental effects on mental health across different population groups (Chan et al. 2024; Badinlou et al. 2024; Solomou and Constantinidou 2020; Ching et al. 2024), suggesting increased demands on services. Similarly, whilst the use of remote appointments has reduced from almost complete remote working at the end of 2020, most services have retained some element of post‐pandemic remote working through a blended approach to service delivery (Zangani et al. 2022).

There is a growing wealth of literature on Telehealth and Telemental health—delivering mental health care via video calls, telephone calls, or text messages (Schlief et al. 2022). Yet although this delivery method is reported as generally acceptable for service users and professionals, a range of impediments to optimal care have been identified (Appleton et al. 2021). Furthermore, different remote delivery methods and implementation strategies are combined within most studies, making specific conclusions difficult to draw (Rains et al. 2022; Appleton et al. 2023). Therefore, whilst a clear picture of mental health service delivery during the pandemic is emerging, evidence on the longer‐term impact is so far lacking. There is a need to understand pre–post pandemic changes in the rate, severity and complexity of mental health referrals, along with the effect of continued remote working, particularly the use of different delivery formats on patient engagement, treatment duration and effectiveness.

CMHNs In the UK deliver care in a tiered service delivery model, working in integrated, multidisciplinary primary care community teams based around general medical practices. This model aims to reduce both the fragmentation of care and the stigma of mental illness, treating people as close to home as possible (National Collaborating Centre for Mental Health 2019). Patients with more severe/complex problems can be ‘stepped‐up’ to specialist secondary care mental health teams providing more intensive residential care. As in most countries, CMHNs working in these UK primary care services were at the front line in dealing with mental distress throughout the pandemic. They have adapted to changes in demand whilst also implementing new strategies for both primary care integration (NHS England 2019a) and digitally enabled care (NHS England 2019b). This study examines the longer‐term impact of the COVID‐19 pandemic on the functioning and effectiveness of community mental health nurses by comparing routine service data from patient cohorts treated before (face‐to‐face), during (remotely) and after (blended) COVID restrictions to assess the effect of delivery format on outcomes and delivery.

## 2 Methods

### 2.1 Design and Setting

Ten CMHNs working in a primary care mental health service delivered assessment appointments from a primary care clinic (the service hub), and treatment appointments across GP practices. The ten CMHNs comprised the total number of mental health nurses in the primary care service, working alongside physical health professionals, such as community and practice nurses, physiotherapists and occupational therapists in integrated primary care teams based around General Practice surgeries, each serving populations of around 4000 to 12 000 patients per practice.

**FIGURE 1:**
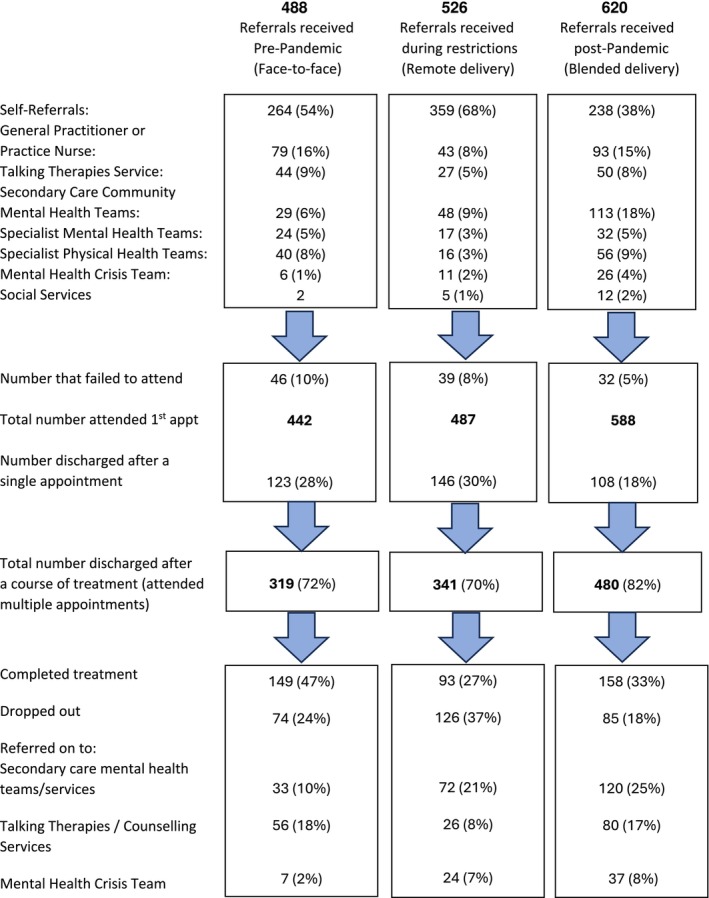
Flow diagram of referrals in three cohorts treated by Primary Care Community Mental Health Nurses.

Referrals were accepted from all mental and physical health professionals, and patients could self‐refer by telephone or online (see Figure 1). At assessment, CMHNs could book patients directly into appointments with other mental health professionals/services depending on suitability, such as therapists and counsellors in primary care (for common mental health problems) or psychiatrist‐led mental health teams in secondary care (for severe and enduring mental illness). This aimed to reduce multiple referrals and assessments between services.

CMHNs delivered treatment for people with a wide range of mental health problems, whose needs could be met by a single mental health professional in primary care. Patients with severe and enduring mental illness such as psychosis or bipolar disorder were treated by a different mental health team under a specialist pathway. This meant that the 10 CMHNs dealt with a high proportion of problems in the non‐psychosis category, such as personality disorders. The nurses delivered a range of evidence‐based interventions for a range of identified mental health problems in line with published guidance (National Institute for Health and Care Excellence 2014a, 2014b, 2015, 2020, 2022, 2023). These included: Dialectical Behaviour Therapy; Solution‐Focused Therapy; Behavioural Activation; Problem‐Solving; Cognitive Behavioural Interventions; Counselling skills and Medication Management. Multiple interventions were often delivered under the description ‘Collaborative Case Management’.

### 2.2 Changes During the Pandemic

Prior to the pandemic restrictions, the CMHNs delivered all appointments face‐to‐face in either primary care clinics, GP practices, or patients' homes. From the implementation of restrictions in March 2000, the CMHNs delivered all appointments remotely, either online by web‐camera or by telephone, until May 2021 when the offer of face‐to‐face appointments resumed. This reinstatement of face‐to‐face delivery was offered alongside the remote appointments via web‐camera—a ‘blended’ approach, which subsequently became a permanent delivery format.

### 2.3 Participants

Three patient cohorts were included in the study:

1. Patient treated face‐to‐face prior to the pandemic (1st April 2019–20th March 2020)
2. Patients treated remotely during the pandemic (23rd March 2020–31st May 2021)
3. Patients offered a blended delivery of face‐to‐face and remote appointments post‐pandemic (1st June 2021–30th Sept 2022).

Each cohort included only patients who started treatment on or after the start date, and whose final appointment occurred prior to, or on the end date. As this is a naturalistic observational cohort study, all patients whose treatment fell within these dates were included in the analysis.

### 2.4 Data Source

The data used was drawn from electronic health records recorded by CMHNs employed by two NHS Trusts working in partnership to deliver the primary care service. Anonymized data was extracted on patient demographics; problem types; treatment type; sessional clinical measure scores; format and number of appointments. All data was extracted, anonymized and subjected to the main analysis within the NHS firewall, and only patients who consented to their anonymized data being used for research purposes were included. At assessment, all patients were given a written and verbal explanation of how their data would be stored, and verbal consent was gained for this to be used anonymously for audit and research purposes. Consent was recorded in the patient's clinical IT system records. Ethical approval was granted by the University Ethics Committee.

### 2.5 Measures

To enable primary care integration the CMHNs used the same clinical IT system as therapists in the NHS ‘Talking therapies’ programme for common mental health problems. This system routinely collected sessional clinical measures of both anxiety and depression symptoms, as well as functioning across a range of areas. The CMHNs did not treat patients with anxiety disorders and depression as the primary problem, but many patients expressed such symptoms as part of their distress, along with functional impairment. Therefore, these self‐report measures were completed by patients via a secure online link emailed to them 48 h prior to each appointment. Patients without internet access or IT literacy completed paper measures prior to their face‐to‐face or telephone appointment. The sessional measures consisted of:

- The Patient Health Questionnaire (PHQ‐9) (Kroenke et al. 2001): A nine‐item self‐report questionnaire evaluating depressive symptoms. Total scores range from 0 (no symptoms) to 27 (severe depressive symptoms).
- The Generalised Anxiety Disorder Questionnaire (GAD‐7) (Spitzer et al. 2006): A seven‐item self‐report questionnaire focusing on symptoms of anxiety. Total scores range from 0 (no anxiety symptoms) to 21 (severe anxiety symptoms).
- The Work and Social Adjustment Scale (WASA) (Mundt et al. 2002), a self‐report questionnaire measuring the impact of a patient's main problem on their ability to function in terms of work, home management, social leisure, private leisure and personal or family relationships. Each item is rated on a 0–8 scale.

These measures have each demonstrated high levels of sensitivity; reliability and consistency (Gilbody et al. 2007; Johnson et al. 2019; Mundt et al. 2002), which has supported their use within the national psychological therapies programme.

### 2.6 Statistical Analysis

All analyses were performed using SPSS 25.0 software, with significance set at 5%. Descriptive analyses were conducted for each cohort to present the means, standard deviations, counts and proportions as appropriate for each variable. Mean pre‐post treatment change on clinical measures was examined using Wilcoxon tests, and effect sizes were calculated using the formula: Pre‐treatment mean − post‐treatment mean divided by pre‐treatment standard deviation.

### 2.7 Definition of ‘Reliable Improvement’

To help examine predictors of outcome, patients were categorised according to whether they achieved, ‘Reliably improvement’ or not. This was achieved if patients were at clinical caseness (≥ 10 on PHQ‐9 and/or ≥ 8 on GAD‐7) at the start of therapy and their post‐treatment scores fell by more than the measurement error of both questionnaires, which is 6 on the PHQ‐9 and 4 on the GAD‐7 (NHS Digital 2019).

Multivariable binomial logistic regression was used to model the association between several key variables and the likelihood of achieving reliable improvement. After adjusting for gender, age and baseline measures (PHQ‐9 and GAD‐7) the variables entered into the regression model included: Format of treatment delivery (Face‐to‐Face or remote); source of referral; presence of a co‐morbid physical health condition and employment status.

## 3 Results

### 3.1 Characteristics of Referrals Before, During and After the Pandemic

Referrals from specialist mental health services increased from 12% to 27% pre‐to‐post pandemic. Conversely, whilst the proportion of self‐referrals increased during the 13 months of UK pandemic restrictions (when many patients avoided using the NHS), self‐referrals subsequently fell to below pre‐pandemic levels (from 54% to 38%) as the proportion of referrals from secondary care mental health services rose in the post‐pandemic period (see Figure 1).

From pre‐to‐post pandemic there were observed increases in referrals with recorded: Comorbid long‐term conditions (36%–41%); medium–high risk status (9%–17%); severe and enduring mental illness (< 1%–8%); somatisation disorder (2%–6%) and complex bereavement or loss (4%–10%). A fall in the proportion of referrals with common mental health problems was observed pre‐to‐post pandemic (23%–17%) (Table 1).

**TABLE 1:** Characteristics of referrals in three cohorts treated by Primary Care Community Mental Health Nurses.

	Referrals assessed Pre‐pandemic *N* = 442	Referrals assessed during restrictions *N* = 487	Referrals assessed post‐pandemic *N* = 588
Mean age (S.D.)	37.4 (15.1)	35.9 (16.2)	38.4 (15.9)
Gender: female/male	293 (66%) / 149 (34%)	326 (67%) / 161 (33%)	369 (63%) / 219 (37%)
Co‐morbid long‐term physical health condition	160 (36%)	194 (40%)	239 (41%)
Co‐morbid disability	77 (17%)	70 (14%)	108 (18%)
Risk rating: total	433	473	566
No risk	58 (13%)	73 (15%)	60 (10%)
Low	333 (77%)	361 (76%)	410 (72%)
Medium	39 (9%)	34 (7%)	69 (12%)
High	3	5 (1%)	27 (5%)
Total problem type recorded (*N*)	442	487	588
Personality problems; complex trauma and/or abuse (past or recent/current)	211 (48%)	226 (46%)	280 (47%)
Severe depression and/or anxiety disorders	105 (23%)	117 (24%)	101 (17%)
Complex bereavement/adjustment disorder	21 (4%)	46 (9%)	62 (10%)
Eating disorders	29 (6%)	28 (6%)	44 (7%)
Somatisation disorder	12 (2%)	25 (5%)	36 (6%)
Drug/alcohol abuse	17 (4%)	10 (2%)	21 (3%)
Bipolar depression	2	9 (1%)	22 (4%)
Psychosis	0	10 (2%)	24 (4%)
Other or not recorded	45 (10%)	16 (3%)	20 (3%)

Mean pre‐treatment severity scores were higher on all clinical measures in the post‐pandemic period compared to pre‐pandemic means (Table 2). Despite this, medium to large effect sizes were observed in mean change scores (E.S. 0.5–0.9, Table 2) during both the pre‐ and post‐pandemic periods. Observed effect sizes were lower during the pandemic restrictions (E.S. 0.1–0.6, Table 2) as a high proportion of patients discontinued treatment in response to the onset of ‘lockdown’.

**TABLE 2:** Mean pre‐ and post‐treatment scores for treatment completers prior to, during, and post pandemic restrictions.

Measure	Pre	Post (SD)	E.S^a^	Pre	Post (SD)	E.S^a^	Pre	Post (SD)	E.S^a^
Work and Social Adjustment Scale									
Work	4.3 (2.6)	2.9 (2.7)	0.5	4.2 (2.6)	3.9 (2.7)	0.1	4.8 (2.5)	3.0 (2.5)	0.7
Home management	3.9 (2.4)	2.5 (2.3)	0.6	4.0 (2.5)	3.0 (2.4)	0.4	4.4 (2.3)	3.2 (2.5)	0.5
Social leisure activities	4.6 (2.5)	3.0 (2.5)	0.6	4.3 (2.5)	3.2 (2.6)	0.4	4.9 (2.3)	3.5 (2.4)	0.6
Private leisure activities	3.7 (2.6)	2.5 (2.3)	0.5	3.8 (2.4)	2.9 (2.3)	0.3	4.2 (2.4)	3.0 (2.5)	0.5
Relationships	4.2 (2.4)	2.7 (2.4)	0.6	4.1 (2.3)	3.6 (2.5)	0.2	4.3 (2.3)	3.1 (2.4)	0.5
PHQ‐9	15.8 (6.2)	10.2 (7.5)	0.9	17.4 (6.6)	13.7 (8.0)	0.5	17.7 (6.1)	12.4 (7.8)	0.8
GAD‐7	13.6 (5.6)	8.6 (6.3)	0.8	14.4 (5.7)	10.9 (6.8)	0.6	14.7 (5.2)	10.2 (6.5)	0.8

^a^ Formula: (Pre‐treatment mean − post‐treatment mean)/pre‐treatment SD; 0.8 upwards is usually regarded as large and clinically significant.

### 3.2 Engagement and Treatment Delivery Before, During and After the Pandemic

The format of treatment delivery changed from 90% face‐to‐face pre‐pandemic to 90% remote delivery during the pandemic restrictions, and then moved to a mixture of 21% face‐to‐face/56% remote/23% blended delivery post‐pandemic (Table 3).

**TABLE 3:** Format and amount of treatment delivery prior to, during, and post‐pandemic restrictions.

Treatment format	Referrals treated pre‐pandemic *N* = 319	Referrals treated during restrictions *N* = 341	Referrals treated post‐pandemic *N* = 480
Face‐to‐face only	287	12	102
Remote—telephone only	13	88	47
Remote—Web‐camera only	0	137	162
Blended—F2F and remote	19	23	111
Blended remote—telephone and web‐camera	0	81	58

Engagement with treatment improved slightly post‐pandemic, with more patients attending treatment beyond the first appointment (72% pre to 82% post‐pandemic). Drop‐outs from treatment increased during the pandemic restrictions (many patients discontinued treatment when ‘lock‐down’ began), but there was a reduction in the drop‐out rate after restrictions lifted (from 24% pre to 18% post‐pandemic). There was a post‐pandemic increase in the proportion of patients referred on to secondary care specialist mental health services (from 12% pre to 33% post‐pandemic, Figure 1).

Drop‐out rates varied by format of treatment delivery with the highest proportion of dropouts occurring in patients who received telephone appointments (69 out of 148; 47%); then telephone blended with web‐camera (52 out of 139; 37%). The dropout proportions were equal between patients who received face‐to‐face appointments (80 out of 401; 20%) and those who had face‐to‐face blended with remote appointments (31 out of 153; 20%). Drop‐out rates were lowest in those patients who had treatment via web‐camera (53 out of 299; 17%). The mean number of treatment appointments patients attended up to discharge increased from 11.1 (S.D. 8.7) before the pandemic to 12.8 (S.D. 9.6) in the post‐pandemic period. This fell to 9.5 (S.D. 7.2) during the Covid restrictions period, which was affected by the number of patients who discontinued treatment early when ‘lock‐down’ was implemented.

### 3.3 Predictors of Reliable Improvement at Discharge

After adjusting for gender, age and baseline measures, the blended delivery method of face‐to‐face and remote appointments was positively associated with reliable improvement at discharge (OR 1.87; 95% CI 1.00 to 2.67) (Table 4). A positive association between face‐to‐face appointments and reliable improvement was observed (OR 1.69; 95% CI 0.91 to 2.62), but this was not significant at the 0.05 level (Table 4). Self‐referral was also positively associated with reliable improvement (OR 2.21; 95% CI 1.22 to 3.15).

**TABLE 4:** Summary of binary logistic regression analysis for variables predicting reliable improvement (all patients pooled).

Variable	OR^a^; 95% CI^b^	*β* ^c^; 95% CI^b^	*p*
Delivery format (*N*)			
Blended: F2F & Remote (193)	1.87; 1.00 to 2.67	0.66; 0.07 to 1.28	0.035
Face to face only (560)	1.69; 0.91 to 2.62	0.61; −0.02 to 1.17	0.059
Web‐camera only (387)	1.24; 0.86 to 2.05	0.38; −0.26 to 1.05	0.152
Web‐camera & telephone (159)	1.09; 0.79 to 1.96	0.35; −0.29 to 1.02	0.179
Telephone only (218)	0.52; 0.26 to 0.87	−0.69; −1.33 to 0.15	0.017
Source of referral			
Self	2.21; 1.22 to 3.15	0.78; 0.19 to 1.26	0.013
Primary Care	1.14; 0.36 to 3.09	0.34; −0.29 to 1.04	0.180
Specialist physical health	1.06; 0.33 to 2.98	0.31; −0.35 to 1.03	0.191
Secondary care MH	0.74; 0.42 to 1.36	−0.39; −1.05 to 0.11	0.077
Co‐morbid long term physical condition	0.88; 0.7 to 1.89	−0.44; −1.22 to 0.15	0.094
Employed	1.19; 0.37 to 2.24	0.37; −0.20 to 1.06	0.173
Unemployed	0.72; 0.43 to 1.45	−0.36; −0.88 to 0.02	0.098

^a^ Odds ratio.

^b^ 95% confidence interval.

^c^ Beta‐coefficient.

Patients who received treatment via telephone were significantly less likely to demonstrate reliable improvement (OR 0.52; 95% CI 0.26 to 0.87). A negative association with reliable improvement was also observed in patients referred from secondary care mental health services (OR 0.74; 95% CI 0.42 to 1.36), but this did not reach significance at the 0.05 level (Table 4).

## 4 Discussion

Ten Community Mental Health Nurses (CMHNs) working in primary care successfully adapted from delivering face‐to‐face treatment pre‐pandemic to remote or blended delivery post‐pandemic, without an observable reduction in improvement rates. To maintain these post‐pandemic outcomes with remote delivery, nurses provided more appointments on average per patient compared to pre‐pandemic (face‐to‐face) delivery. This may be due to the increased severity and complexity of post‐pandemic referrals, as a larger proportion came from secondary care mental health teams with higher risk ratings. This increased severity/complexity remained at 16 months post‐pandemic and is compatible with the evidence that post‐pandemic mental distress across populations has remained at higher levels than those observed pre‐pandemic (World Health Organisation 2022).

The increased number of required CMHN appointments appeared to be offset by increased efficiencies from remote working, as there was no reduction in patient throughput or numbers treated post‐pandemic. In addition to secure online video calls, the service used a clinical IT system that prompted patients to enter clinical measures directly onto the system through a secure email link sent prior to each appointment. The use of such time‐saving technology is central to transforming the UK's National Health Service (Department of Health and Social Care 2021), and the interoperability of such clinical IT systems is essential for delivering integrated care (NHS England 2019b).

The use of Web‐camera appointments blended with face‐to‐face appointments predicted better outcomes and was comparable to face‐to‐face delivery alone. These positive outcomes are comparable to those reported in systematic reviews of the evidence on remote treatment for a range of mental health problems (Schlief et al. 2022; Farrell et al. 2022; Varker et al. 2019). Blended delivery allows for both the therapeutic effects of visual, non‐verbal communication and the convenience of remote delivery. The importance of patient convenience for engagement is supported by web‐camera delivery showing the lowest drop‐out rates.

The finding that telephone was inferior to online video appointments is not a commonly reported observation across studies of remote treatment. Telephone appointments have been reported to have no negative impact on therapy for common mental health problems (Saxon et al. 2023); reviews have recommended offering a choice of modality (Appleton et al. 2023; Schlief et al. 2022) and surveys have reported that community mental health professionals view it as beneficial (AlRasheed et al. 2022; Rains et al. 2022). This may be due in part to over‐inclusivity/lack of clarity in the definition of remote delivery within the literature. The commonly used term ‘Telecare’ has been defined as the delivery of health care directly to users in their own homes, ‘Supported by information and communication technologies such as telephone, videoconferencing, and applications’ (The Audit Commission 2004). Many studies and reviews have included a mixture of these formats, often preventing a direct comparison between them.

Studies that have reported positive outcomes from telephone‐delivered interventions have tended to use telephone calls in a specific way to support the use of guided self‐help workbooks (Turner et al. 2014; Lovell et al. 2006) or online computer programs (Španiel et al. 2012), often within a collaborative care model for less severe common mental health problems (Capobianco et al. 2023). In contrast, many of the problems treated by the CMHNs were of a more severe/complex type. Web‐camera appointments still allow for visual non‐verbal communication (albeit with restrictions, Connolly et al. 2020) whereas telephone appointments do not. Mehrabian (1972) originally claimed that 55% of communication is body language. Such a static figure may be misleading as non‐verbal communication constantly modulates the effect of verbal speech in therapeutic communication, and so cannot be separated from it (Del Giacco et al. 2020). It has been nearly 50 years since Egan (1975) introduced his acronym SOLER as an aid for teaching non‐verbal communication, and mental health nurses have long been taught that non‐verbal skills are essential to achieving the congruence, understanding, and empathy that are the core elements of the person‐centred recovery model of mental health nursing (Shanley and Jubb‐Shanley 2007; Cusack et al. 2017).

The CMHNs delivered direct therapeutic interventions such as Dialectical Behaviour Therapy (DBT) and Person‐centred Counselling during telephone appointments, rather than supporting the use of self‐help materials/online programmes. A key feature of DBT is helping service users learn how to helpfully interpret non‐verbal communication such as facial expressions (reducing misinterpretation, Linehan 2015). Such skills practice is not possible via telephone, and individuals are less accurate in perceiving such behaviours in phone interactions than in‐person interactions (Sadikaj and Moskowitz 2018). In person‐centred counselling, non‐verbal communication is essential to convey congruence, empathy, and positive regard (Rogers 1986), yet it is more challenging for mental health professionals to understand and convey emotional information in telephone calls (Turgoose et al. 2018), which can also limit the options for emotional expression (Parkinson 2008). Therefore, the negative impact of telephone appointments on outcomes in this study questions the suitability of this format to deliver lengthy, intensive therapy appointments with CMHNs.

The outcomes from telephone delivery may have been negatively impacted by a proportion of patients who initially switched to this format during the first ‘lockdown’ and subsequently disengaged because they could not access online video appointments. This highlights a potential inequity in remote delivery, as some patients do not possess web‐camera devices; internet access or the IT skills to use them. In the UK, as in many countries, Digital exclusion—when people may not have the skills, confidence, motivation or ability to connect to the internet (NHS England 2024), disproportionately affects marginalised groups such those with severe mental illness (Tobitt and Percival 2019); older people; people in lower income groups; the unemployed; those in social housing; people with disabilities; those with fewer educational qualifications; homeless people and people whose first language is not English (UK Government, 2017). Digital inclusion strategies (e.g., where service users are loaned tablets) can improve access to tele‐mental health services (Oliver et al. 2024) and therefore could be built‐into community mental health services that routinely offer remote access.

47% of patients had a personality disorder or complex trauma recorded as their problem descriptor. The delivery of DBT via video‐conference for patients with borderline personality disorder (BPD) has previously found no impact on engagement compared to face‐to‐face appointments (Walton et al. 2023). However, when telehealth sessions for patients with BPD were delivered either by phone, video‐conference, or a combination of the two, some patients reported more difficulties in controlling distressing emotions. These included anger and impulses to self‐harm, as well as difficulties in establishing and maintaining agreed treatment boundaries, which increased risk (Heidari et al. 2023). The negative impact of telephone appointments on engagement and outcomes in this study adds to the concern about the broader utility and effectiveness of telephone appointments with this patient group. This therefore emphasises the importance of addressing digital exclusion for patients with BPD.

The proportion of post‐pandemic patients referred on to the ‘Talking therapies’ service (17%) remained similar to pre‐pandemic levels (18%), whereas referrals to secondary care mental health services increased from 10% to 25%. This may reflect the increased need for CMHNs to complete stabilisation work with patients whose lives are initially too chaotic to engage with structured support from psychiatrist‐led mental health teams, or psychological therapists.

## 5 Strengths and Limitations

This is the first detailed retrospective examination of the impact of remote working on the clinical effectiveness of CMHNs over a longer‐term period of routine practice after the pandemic. The dataset comprising 1634 referrals across the 3 cohorts before, during and after the pandemic period provides a comprehensive view of service changes since the implementation of remote working. The primary care CMHNs shared the same clinical IT system used by the therapists in the UK's ‘Talking Therapies’ service, which records detailed problem descriptors and routinely collects sessional outcome data. Therefore, the data is more detailed than in the usual IT systems of many community mental health services.

The main limitation of the study relates to the amount of missing outcome data for post‐treatment scores on the clinical measures. Out of 1140 patients who completed a course of treatment by attending 2 or more appointments, 176 (15%) had only one set of scores recorded (assessment). The majority of these dropped out of treatment (107 out of 285 who dropped‐out were missing scores). Where patients who dropped out had multiple scores recorded, the last scores entered were taken as their final outcomes. These 176 patients were not included in the final analysis, and so caution should be employed in drawing conclusions from the reported effect sizes. Despite this, the service achieved 85% complete data for all patients treated, which compares favourably to the 38% data completeness reported in a previous observational study of routine primary care mental health services (Stiles et al. 2007).

Another limitation arises from the clinical measures used in the service. Although the PHQ‐9, GAD‐7 and WSA are reliable measures for assessing the symptoms of common mental health problems, they do not capture/measure other symptoms such as emotional dysregulation or indicators of chaotic lifestyles in patients with more complex problems. Therefore, there is a need for an increased use of disorder‐specific measures in future CMHN treatment and research. This should be complemented by qualitative studies to capture the views/experiences of service users and explore what aspects of care they receive from Community Mental Health Nurses they find most helpful or detrimental to their recovery.

## 6 Conclusions

Community Mental Health Nurses maintained clinical effectiveness as they moved to remote working, despite an increase in the severity and complexity of referrals up to 16 months after the pandemic. Whilst this increased the amount of patient time/appointments required, this was enabled through the efficient use of remote appointments, which quickly became embedded alongside face‐to‐face delivery as routine practice. The flexible use of online video appointments predicted better engagement and outcomes. Yet delivering all therapeutic interventions by telephone appeared detrimental to engagement with CMHNs, predicting lower rates of improvement for patients.

## 7 Relevance for Clinical Practice

Referrals to Community Mental Health Nurses in primary care appear to have remained more severe/complex for a significant duration due to the fall‐out from the pandemic. CMHNs can cope with the increased intensity/demand by improving efficiencies through the use of technology that supports remote working. This includes clinical IT systems that can take over administrative tasks such as scheduling appointments, sending text reminders and collecting clinical ratings from patients prior to appointments.

The outcome data from this study/service suggests that the flexible use of online video appointments offered as a choice for patients can continue to help CMHNs deliver effective therapeutic interventions whilst also improving attendance/engagement. This data also suggests that CMHNs should consider limiting the use of telephone appointments to briefer calls aimed at supporting less severe patients to use self‐help materials/programs, or to monitor/prompt medication compliance, rather than attempting full therapeutic sessions for complex problems by phone.

## Conflicts of Interest

The authors declare no conflicts of interest.

## Data Availability

The data that support the findings of this study are available on request from the corresponding author. The data are not publicly available due to privacy or ethical restrictions.
